# *Scotty*: lattice coincidences for macromolecular crystallographic phasing

**DOI:** 10.1107/S2059798326005711

**Published:** 2026-06-24

**Authors:** Airlie J. McCoy, Lawrence C. Andrews, Herbert J. Bernstein, Randy J. Read

**Affiliations:** a Cambridge Institute for Medical Research, Department of Haematology University of Cambridge The Keith Peters Building, Hills Road CambridgeCB2 0XY United Kingdom; b Ronin Institute for Independent Scholarship 2.0 9515 NE 137th Street Kirkland WA98034 USA; c Fresh Pond Research Institute c/o Brookhaven National Laboratory NSLS II Upton NY11973 USA; Lund University, Sweden

**Keywords:** non-isomorphism, Niggli cell, crystallographic contaminants

## Abstract

When a macromolecular crystal lattice targeted for study is closely related to a previously studied crystal lattice, phasing the target crystal by difference (fast-)Fourier transform (DFFT) methods is preferable to performing molecular replacement. The application *Scotty*, within the *Phasertng* codebase, is software for the identification of coincident lattices and downstream processing. All crystallographic PDB entries are organized into a scikit-learn ‘BallTree’ index under the Niggli cell distance metric ‘NCDist’. Nearest neighbours under the metric are progressed to test structure-factor intensity correlation. The structure from the lattice with the highest correlation with the target is used to phase the target lattice, and the coordinates are taken forward to coordinate refinement using a wide convergence radius protocol. The method can identify lattice coincidences accounting for very significant non-isomorphism. The nearest-neighbour search is space-group agnostic, so that coincident lattices are identified even when the nominal space groups are different, the symmetry of one lattice being described as a subgroup of the other or a higher metric symmetry.

## Abstract 2

*Scotty* is a tool that leverages Niggli-cell matches to identify similar crystallographic lattices and perform phasing with difference Fourier methods.

## 1. Introduction

That two crystallographic lattices built from the same macromolecule(s) are coincident is not necessarily immediately obvious. There may be overt differences in the unit-cell settings and/or symmetry operators assigned to the diffraction pattern (due to software differences, poor data quality, incompleteness or twinning), and these differences may make the lattices appear distinct at first glance. More subtly, differences in crystallization conditions, bound ligands, temperature, crystal handling or cryocooling, which arise particularly when experiments are performed by different crystallo­graphers separated by time and laboratory, can lead to perturbations in packing and discrepancies in the space group and unit-cell dimensions, leading to differences that may obscure the underlying similarity.

The identification of any previously known lattices that are coincident with a target structure at the start of crystallo­graphic investigation allows phasing by difference (fast-)Fourier transform (DFFT) methods, which is preferable to performing molecular replacement. DFFT phasing has five major advantages: (i) it immediately resolves any ambiguity in Laue symmetry or space-group screw symmetry, (ii) it ensures matching of origins and space-group settings between related structures, (iii) it facilitates comparison of the structures by ensuring that the coordinates of reference/deposited molecules, chosen from all the symmetry-equivalent options in the two lattices, superimpose without further organization, (iv) it trivializes phasing and (v) it highlights convergences of quaternary structure within the lattice that may be of biological relevance.

Coincident lattices can be identified from the crystallo­graphic data alone, unbiased by any preconceived idea of the macromolecular contents of the crystal. Unexpected, accidental contaminant crystallization may be revealed in a broad screen of all PDB structures (Hungler *et al.*, 2016 ▸), provided that the crystal form of the contaminant has previously been determined and deposited in the PDB archive, which is a significant caveat given that many contaminants are intrinsically prone to crystallize easily in novel or nondeposited crystal forms (Rankin & Smith, 2025 ▸; Hungler *et al.*, 2016 ▸).

DFFT phasing methods should be contrasted with molecular replacement. Molecular replacement is applied for a novel crystal form when a model structure is available, while DFFT methods are used for detecting electron-density differences between closely related crystal structures, one already phased. The conceptual overlap in using known structural information to infer new information (as opposed to experimental phasing methods) has led to many PDB entries being mistakenly annotated as ‘phased by molecular replacement’ when a more accurate description would be ’phased by DFFT methods’. Although identifying and correcting historical annotations in a PDB-wide remediation is impossible, our development of specialist software for DFFT phasing may help mitigate this problem going forward.

DFFT phasing is standard for identifying ligand-binding sites in high-throughput drug-screening campaigns. An initial ‘reference’ crystal (often the apoenzyme) is isomorphous to a series of ligand-soaked crystals: a DFFT map will reveal bound fragments or inhibitors. The *Pan-Dataset Density Analysis* (*PanDDA*) method has been developed to greatly improve sensitivity for detecting low-occupancy ligands by statistically comparing electron density across large ensembles of apo and ligand-bound structures, thereby revealing subtle binding events that are often obscured in individual DFFT maps (Pearce *et al.*, 2017 ▸). DFFT phasing is also used for mutation studies since point mutations and small insertions and deletions that maintain the overall fold commonly do not disturb the lattice.

There are currently few tools for identifying coincident lattices, and all have critical deficiencies for the task at hand. *SIMBAD* performs a lattice search against a precomputed PDB-derived database of Niggli cells (defined below), selecting candidate entries whose Niggli cell parameters fall within fractional tolerances and ranking them by cell-parameter and volume differences before testing the retained models by molecular replacement (Simpkin *et al.*, 2018 ▸); this method is limited by the discontinuities in metric space for Niggli cells, as discussed in detail below. The Bilbao Crystallographic Server is a conceptually related resource for evaluating space-group symmetry and subgroup lattice relationships: it has a web-based interface and does not have a database search (Aroyo *et al.*, 2006 ▸). The European Bio­informatics Institute has a Niggli cell range-based search tool accessible by API, but is also limited by the discontinuities in metric space for Niggli cells (Armstrong *et al.*, 2020 ▸). The *SAUC* server (*Search for Alternative Unit Cells*; McGill *et al.*, 2014 ▸) matches query lattices to those in the PDB (Berman *et al.*, 2003 ▸) and COD (Crystallography Open Database; Gražulis *et al.*, 2012 ▸) through a web interface of manually entered unit-cell parameters using a number of different reduced cell distance metrics, including Niggli cell distance metrics. Of these, only *SIMBAD* carries any lattice matches forward to phasing, although not by DFFT.

The aim of our work is to improve and streamline the identification of lattice coincidences accounting for very significant non-isomorphism, even when the nominal space groups of the coincident lattices are different, and perform DFFT phasing. Apart from the technical advantages, this work should support the study of lattice associations for biological relevance more broadly (McCoy *et al.*, 2026 ▸).

## 2. Background

We use the term ‘coincident’ lattice to describe lattices of structurally similar (homologous) macromolecular components with similar spatial organization (*i.e.* their translational and rotational symmetries) and crystal-packing interactions.

In crystallographic literature, the terms ‘isomorphous’ and ‘non-isomorphous’ are used in a variety of contexts. Crystals may be termed ‘non-isomorphous’ if there is a change in the unit-cell dimensions, as may occur during cryocooling or radiation damage; percentage cell-length changes or cell-volume changes are often used to measure the degree of non-isomorphism. In experimental phasing, ‘non-isomorphous’ may refer to heavy-atom-soaked crystals that cannot provide much useful experimental ‘isomorphous’ phasing information. ‘Non-isomorphism’ may also describe space-group changes driven by metastable lattice states, where small perturbations, such as ligand soaking or temperature change, permit a transition between closely related symmetry arrangements. In multi-crystal data collection, the definition of ‘isomorphism’ is often whether structure-factor intensities can be successfully merged in data processing without a substantial deterioration in merging statistics. Many of these designations of ‘isomorphous’ versus ‘non-isomorphous’ are implicitly tied to the resolution of comparison.

If ‘isomorphous’ and ‘non-isomorphous’ are binary classifiers in any given context, we use the term ‘lattice coincidence’ to encompass both, to the limit of a detectable correlation between binned, normalized structure-factor intensities between matched Miller indices for all data up to 3 Å resolution (see below).

### 2.1. Niggli cell

Searching the database for coincident lattices starts with a search for unit-cell coincidences. Since a lattice can be represented equivalently in uncountably many unit-cell definitions, a canonical form, the Niggli cell, is commonly used to reduce any unit cell to a unique, standardized description. The Niggli cell reduction transforms any choice of primitive vectors into a unique reduced cell that satisfies *a* ≤ *b* ≤ *c*, α ≤ β ≤ γ, and the off-diagonal metric dot products obey additional inequalities that ensure that the cell is as small and as close to orthogonal as possible (see the supporting information; Niggli, 1928 ▸).

Although the Niggli cell is unique for a given set of unit-cell parameters, small perturbations can cause the defining inequalities to flip, leading to large discontinuous changes in the Niggli cell at boundaries, moving into a distant region of parameter space. Lattices with similar unit-cell axis lengths and angles close to 90° are particularly prone to these instabilities, and these lattices are common in macromolecular crystallography (Andrews *et al.*, 1980 ▸).

Note that symmetry operators describing the intensity relationships of the reciprocal lattice are irrelevant to the Niggli cell reduction.

### 2.2. Niggli cell server at PDBe

The European Bioinformatics Institute (EBI), through its Protein Data Bank in Europe (PDBe) interface, provides a search tool for Niggli-reduced unit cells (Armstrong *et al.*, 2020 ▸). This service allows users to identify crystallographic entries with similar lattice parameters in the PDB by defining search regions as axis-aligned boxes surrounding a reference Niggli cell. While this method offers an efficient, and PDB database-synchronized, way to locate related lattices, it is limited in its ability to find coincident lattices because of the instability of the Niggli reduction near boundary regions of the reduced-cell domain. As a result, some coincident lattices will fall outside the chosen search box and be missed by purely box-based Niggli searches. A more comprehensive search strategy would identify ‘nearest-neighbour’ lattices using a Niggli cell distance metric.

### 2.3. NCDist

The distance between lattices is quantified using various Niggli-cell metrics (Andrews & Bernstein, 2014 ▸), or indeed other metrics based on other unit-cell reductions (Andrews *et al.*, 2019 ▸; Bernstein *et al.*, 2023 ▸). We use the NCDist (Niggli cone embedding distance; Andrews & Bernstein, 2014 ▸) for comparisons. The NCDist metric is defined in a six-dimensional space **G**^6^,
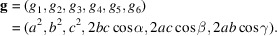


NCDist is calculated from the **G**^6^ Euclidean distance (units of Å^2^) by taking the square root, thereby linearizing the metric to units of Å, and then multiplying by a scale factor to put the value in the same order of magnitude as the associated differences in unit-cell edge lengths. Different implementations choose different factors, which may depend on the cell dimensions (Andrews & Bernstein, 2014 ▸). We use the constant scale factor 0.1 to be consistent with the *SAUC* implementation (see below). Adopting a single metric across different software platforms provides clear advantages for crystallo­graphers, enabling a straightforward comparison of results.

### 2.4. *SAUC* server

The *SAUC* server (*Search for Alternative Unit Cells*; McGill *et al.*, 2014 ▸) is a powerful tool for matching query lattices to those in the PDB and COD. *SAUC *organizes the Niggli-cell **g** vectors as a ‘NearTree’ metric index, which is designed to efficiently compare tens of thousands of entries and rapidly identify the nearest neighbours in **G**^6^ metric space. Individual queries are made through a web form. The *SAUC* server reports NCDist calculated with a scale factor of 0.1, which puts NCDist in the range of the related differences in unit-cell edge distances for macromolecular structures (around 100 Å).

### 2.5. Minimum NCDist

Finding the minimal NCDist between two lattices requires taking account of Niggli-cell instabilities at inequality boundaries, which in practice can only be performed for a limited combinatorial depth. Due to this computational limitation, no current algorithm is guaranteed to find the minimum of NCDist for all combinations of Niggli-cell pairs. Current algorithms optimize for physically reasonable lattices of real molecules, excluding unphysical extremes. The challenge of finding the minimum NCDist becomes increasingly pronounced as the distance between two lattices increases, owing to the concomitant rise in the number of boundary crossings that must be evaluated.

We optimized the implementation of NCDist used in *SAUC*, achieving a two order-of-magnitude improvement in computational speed (see Appendix *A*[App appa]), which removed significant bottlenecks caused by NCDist calculation for generation of the NCDist metric-space index of the PDB and subsequent querying of the index.

### 2.6. Intensity correlation

Low NCDist values are sufficient for matching geometric lattices, but are not sufficient for proving the matching of macromolecular crystals.

Structure-factor intensity correlation (CC_*I*_) is a robust method for comparing crystallographic datasets (Karplus & Diederichs, 2012 ▸; Diederichs & Karplus, 2013 ▸). It can be calculated without phases, a prerequisite for the analysis to be performed prior to phasing. Data must be normalized in resolution shells for calculation of CC_*I*_, without which CC_*I*_ can be misleadingly high. Data should also be expanded to *P*1, so that data with space-group differences and/or reduced to different unique reciprocal-space volumes may be compared.

All allowed data re-indexing operations must be considered during the CC_*I*_ comparison. Re-indexing operators are linear transformations defined by a unimodal matrix which map the Miller indices to a transformed set of Miller indices preserving the lattice translation symmetry, unit-cell volume and handedness. They are enumerated for each space group. Re-indexing operators account for crystallographic differences associated with axis permutations, obverse/reverse rhombohedral settings, lattice rotations in tetragonal and hexagonal systems, and nonstandard monoclinic space-group choices.

In crystallographic maximum-likelihood theory, σ_A_ is the complex correlation between true and model structure factors, and the expected real-space map correlation. √CC_*I*_ (= 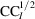) is an estimate of σ_A_ (Hauptman, 1982 ▸; Read, 1986 ▸) and hence is a more interpretable metric of lattice similarity and isomorphism than CC_*I*_ itself. Hence, reporting and decision making is based on √CC_*I*_.

### 2.7. Intensity correlation and resampling differences

Changes in the unit-cell parameters cause the molecular transform to be sampled differently. Resampling differences, and the degree of consequent changes in √CC_*I*_, depend not on the percentage change in cell edges (a common misconception; K. Cowtan, personal communication) but on the absolute difference. If two cells differ by δ Å along a given dimension, then the structure factors along that direction become uncorrelated at δ Å regardless of the total cell length.

**Figure 1:**
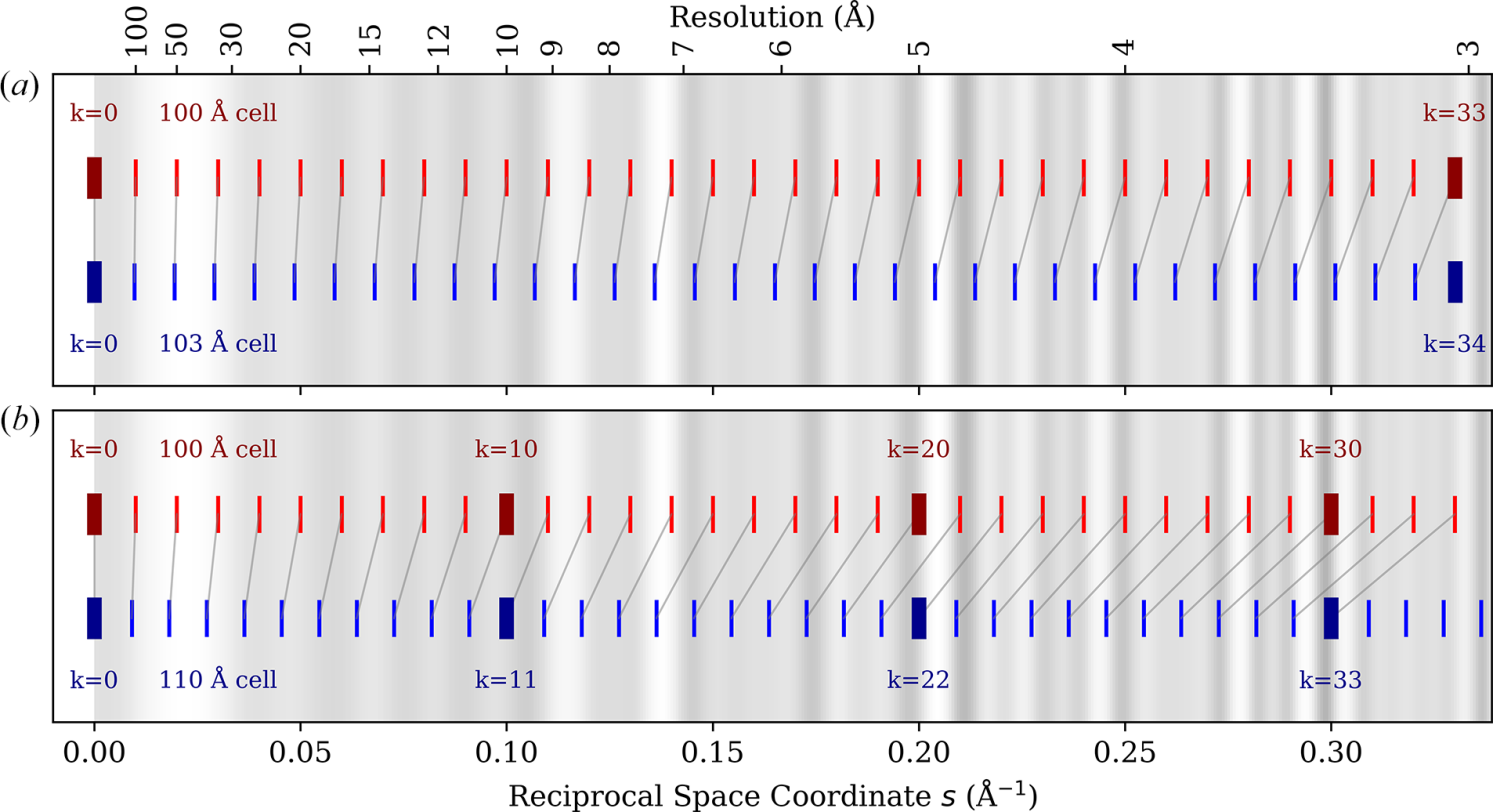
Isomorphism and sampling of the molecular transform. Reciprocal-lattice point positions for pairs of unit cells with differing unit-cell parameters are compared. Each panel shows two sets of reciprocal-space lattices for unit cells of lengths 100 Å and (*a*) 103 Å or (*b*) 110 Å. Grey shading indicates the molecular transform. Thicker lines highlight positions where lattice points sample the same part of the molecular transform, with labels indicating their indices. The panels illustrate how non-isomorphism alters the sampling of the molecular transform and consequently the correlation between intensities at matched reciprocal-lattice indices.

Fig. 1 ▸ illustrates the effect of non-isomorphism on the sampling of the molecular transform for cells with lengths of 100 Å and (100 + δ) Å. For δ = 3, reflection intensities remain well correlated between matched Miller indices to 6 Å resolution and partly correlated to 3 Å. Values of √CC_*I*_ calculated in resolution bins at resolutions higher than 6 Å will begin to degrade due to the diminishing correlation in intensities in the direction of the corresponding unit-cell reciprocal-lattice vector. For δ = 10, the higher non-isomorphism means that at all resolutions higher than 10 Å there will be no positive contribution to √CC_*I*_ from reflections with reciprocal-space vectors in the direction of the non-isomorphous axis of the reciprocal lattice.

## 3. Implementation

Our software *Scotty* within the *Phasertng* codebase (McCoy *et al.*, 2021 ▸) automates the identification and DFFT phasing of isomorphous structures within the PDB archive using only the integrated, scaled and merged crystallographic data and optionally, the sequence of the (expected) biological unit, which is used only for speed optimization.

### 3.1. Metric-space index

We generated a metric-space index of PDB-deposited structure-associated unit cells based on the ideas underpinning the *SAUC* server but adapted to generate a bespoke data structure suitable for distribution (as a Python ‘pickle’ file) with our software. A bespoke metric-space index has the advantage that it allows us to prepare the data in a format suitable for our specific requirements

In preparing the metric-space index, we associate metadata with each entry for identification and rapid downstream analysis. Metadata extracted from the PDB archive were identifier (‘pdb_id’), title, space group, unit cell, deposition group number (if any) and the sequence of the longest polymer within the asymmetric unit (the ‘primary’ sequence) and its sequence type (protein or nucleic acid). Metadata extracted from *PDB_REDO* (Joosten *et al.*, 2014 ▸), from the summary file available at https://pdb-redo.eu/pdb-redo/others/alldata.txt), were a flag for whether the *R* factor could be reproduced and the resolution of the data used for *R*-factor reproduction (‘URESO’).

Each entry also listed duplicates (as a list of PDB identifiers) in the metadata. Duplicates of every PDB entry were identified to allow trivially isomorphous duplicates of matched entries to be skipped in later analysis, for example skipping nearly identical HEWL structures after a first HEWL match. Crystals were annotated as duplicates if they contained the same macromolecules and had identical deposited space groups, very similar unit-cell parameters (within small absolute tolerances) and an r.m.s.d. of less than 2 Å over all atoms in deposited coordinates without rigid-body fitting (see Appendix *B*[App appb]). Although most PDB entries are singletons by these strict conditions, some PDB entries have hundreds of duplicates.

### 3.2. Computations

All computations were performed on a dedicated Linux workstation running Ubuntu 22.04.5 LTS. The system was equipped with a single AMD Ryzen Threadripper 7980X processor (64 physical cores, 128 hardware threads) operating on an x86_64 architecture, with a maximum clock frequency of 5.37 GHz. The machine provided 125 GB of system memory.

### 3.3. Metric-space index generation

The generation of the BallTree NCDist metric-space index of the Protein Data Bank was undertaken on 10th January 2026 for the 20260101 PDB snapshot: there were 190 770 entries.

The Python structure of the combined ‘BallTree’ metric-index of the PDB and metadata was written to disk as a Python pickle file of approximately 50 Mbytes. We call this Python structure the ‘PDB NCDist metric-space index’.

## 4. Querying

The PDB NCDist metric-space index is loaded from the pickle file. The ‘BallTree’ *query_radius* search algorithm finds all NCDist nearest neighbours, without requiring exhaustive pairwise comparisons. Matches are returned in NCDist order, lowest to highest.

The default radius for search is NCDist = 5 Å and can be altered by user request. Since the linear metric NCDist is calibrated to reflect related unit-cell differences for macromolecular structures (see above), an NCDist of 5 Å is a generous value catching all lattices that are isomorphous to changes of the order of approximately 5–10 Å in one cell dimension, at the limits of useful isomorphism. Lower values, for example 3 Å, can be used to consider only more isomorphous matches.

### 4.1. Input formats

*Scotty* takes a merged MTZ-format reflection file as minimal input. The (expected) biological unit may be entered using a multi-sequence FASTA file, in which each molecule is represented as a separate FASTA entry separated by standard FASTA headers.

*Scotty* should be used in a sequence-agnostic way if there is concern that the crystal may be of a ‘contaminant’ or when performing a contaminant screen.

### 4.2. Forcing matches

A ‘force match’ mode allows the user-directed selection of a particular PDB identifier. The PDB identifier must appear in the returned list from *query_radius*.

Alternatively, the user may provide coordinates (in PDB format, with compulsory CRYST1 card for crystal symmetry), allowing the user to match to coordinates that do not appear in the PDB NCDist metric-space index.

### 4.3. Intensity correlation filtering

√CC_*I*_ is calculated for NCDist matches from either observed or calculated data, depending on the matched entry, with the choice being determined by the metadata stored in the PDB NCDist metric-space index.

Observed structure-factor intensities (or squared structure-factor amplitudes) are used when the metadata stored in the index record that *PDB_REDO* was able to reproduce the *R* factor and that the resolution extended to that for the √CC_*I*_ calculation. Many PDB entries have multiple datasets available for each entry, in which case all datasets are used for the comparison.

Where the metadata indicated that *PDB_REDO* found the PDB-deposited data problematic, intensities are calculated from the model using *phenix.fmodel*.

Observed structure-factor intensities or model coordinates are obtained on the fly by interrogating the PDB. Alternatively, the software can be directed to access a local download of PDB entries under the PDB’s ‘divided’ format, where entries are organized into directories according to the middle two characters of the PDB identifier, under subdirectories ‘pdb’, ‘mmCIF’ and ‘structure_factors’ of the master directory.

By default, the √CC_*I*_ for attempting DFFT phasing is 0.4. Values of √CC_*I*_ over 0.4 correspond to a weighted mean phase error (wMPE) of less than 80° (Rodríguez *et al.*, 2009 ▸), where the weighting skews the value towards the phase error for the high-amplitude reflections that dominate map calculation. A wMPE of 80° is high but nonrandom (with 90° being random), and can be sufficient to develop refined structures for complete models after rigid-body refinement, although at the limit it may require density modification and model building (Millán *et al.*, 2018 ▸).

Lower values, down to 0.2, can be used to consider more non-isomorphous matches. A global map correlation of 0.2 has been used as a lower credibility threshold in large-scale molecular-replacement benchmarking (Oeffner *et al.*, 2013 ▸; Hatti *et al.*, 2020 ▸). Since √CC_*I*_ is an estimate of σ_A_, and σ_A_ is an estimate of the expected real-space map correlation, a √CC_*I*_ of 0.2 should be interpreted as weak but detectable evidence for a coincident lattice, rather than as definitive proof of correctness.

The default resolution for √CC_*I*_ calculation is 3 Å, the resolution at which the parameter-to-data ratio generally allows restrained atomic coordinate refinement for macromolecular structures. If the input data extend to lower resolution, then the comparison is made at the maximum resolution of the input data. The resolution can be altered by user request.

### 4.4. Wide convergence radius refinement

The relative orientation and position of the macromolecules in the asymmetric unit may require adjustments beyond the radius of convergence of coordinate refinement algorithms. *Phasertng* implements a robust rigid-body refinement protocol, normally deployed during molecular replacement, but repurposed in *Scotty* for wide radius convergence refinement in cases of non-isomorphism. The chains in the matched structure are treated as separate rigid bodies, and rigid-body refinement is performed to convergence in a set of sequential rigid-body refinements where the resolution is extended in 1 Å steps, starting at 6 Å, until the resolution limit of the target data.

Prior to rigid-body refinement, the target reflections are re-indexed to the space group and unit cell of the matched structure. There is no change of origin or rearrangement of components within the asymmetric unit when the matched model is placed within the target crystal lattice. The model is prepared for rigid-body refinement and phasing as in standard molecular replacement in *Phaser* (McCoy *et al.*, 2007 ▸), which involves the removal of ordered solvent and using only the isotropic component of the temperature factors, since ordered solvent and anisotropic temperature factors are specific to the crystal form.

### 4.5. Speed optimizations

#### 4.5.1. Sequence filtering

Sequence filtering serves as a speed optimization. When a sequence for the probe is available, *Scotty* applies sequence-based filtering before performing the expensive structure-factor and √CC_*I*_ calculation.

Excluding unrelated macromolecules by sequence can filter out a substantial fraction of the candidates. Protein crystal structures tend to cluster in **G**^6^ space because of biases in space-group occurrence and the relatively uniform sizes of protein domains, so that Niggli cells are not evenly distributed in **G**^6^. The fraction filtered by sequence will be particularly high if the radius encompasses lattices of a structure that has been extensively investigated in ligand-binding studies.

The sequence of the longest polymer in the asymmetric unit (the ‘primary’ sequence) is provided in the metadata returned with the nearest neighbours. Each chain of the probe sequence is compared for sequence identity in turn against the ‘primary’ sequence and the highest sequence identity is used for the filtering. Note that only the ‘primary’ sequence of the entry (protein or nucleic acid) in the index is used for comparison. This normally represents the dominant scatterer. The query may pass the sequence-identity test with the index entry despite containing more or fewer protein or nucleic acid components, generating a false positive. False positives continue to √CC_*I*_ calculation, where they are eliminated. By not exhaustively testing for a complete sequence match, we balance speed against specificity, and prevent mismatches in minor scattering components, such as peptide ligands, from filtering out potential matches.

Searches that produce no matches may have failed due to false negatives, although low sequence identities are unlikely to facilitate DFFT phasing. False negatives can be rescued by repeating the search without the sequence filtering.

The pairwise sequence alignment is performed using the *Biopython PairwiseAligner* in global alignment mode.

By default, one of the target biological unit sequences must have a pairwise sequence identity of at least 30% to the match primary sequence. Sequence identity for filtering can be altered by user request.

#### 4.5.2. Duplicate skipping

Duplicate skipping is a second speed optimization. Nearest neighbours to probes are returned from the query in NCDist order: nearest first. Duplicates of returned candidates, stored as metadata during the build of the PDB NCDist metric-space index, that appear later in the returned list of nearest neighbours are excluded from consideration. In cases where the duplicate list extends to hundreds of structures, this is a very significant reduction in computation.

Although one of the later duplicates could have a higher √CC_*I*_ despite having a higher NCDist value, we assume that our restrictive definition of a duplicate will mean that any difference will not be significant for the purposes of phasing.

Duplicate skipping can be turned off by user request.

## 5. Test cases

### 5.1. Prorenin

There are three structures of prorenin deposited in the PDB from our laboratory (Yan, 2017 ▸): human (PDB entry 4amt), rat (PDB entry 5mlg) and mouse (PDB entry 5mkt). Pairwise sequence identities were 66% for human–rat, 69% for human–mouse and 84% for mouse–rat. The structures were originally solved by molecular replacement using mature human renin as the search model. All three proteins crystallized in the less common space group *I*4_1_, but with a 7 Å variation in the *c*-axis length. Using the human structure as the probe and maximum NCDist = 5 Å, *Scotty* was able to identify the mouse and rat lattices in the PDB archive and correctly place the human structure within the rat and mouse lattices, despite the non-isomorphism (Table 1 ▸). The run time, including rigid-body refinement, was 8 s with sequence filtering and 105 s without sequence filtering.

Large differences (such as 7 Å) in a cell length do not exclude the match from being within the radius of convergence of rigid-body refinement because the distances between the centres of mass of the crystallized macromolecules will likely be less than the change. Unit-cell changes of this magnitude likely arise from cumulative, smaller, translations across the unit cell and may be contributed to by small rotations of nonspherical components. Rigid-body refinement can move components to their correct poses because at least some internal molecular coordinates require shifts much less than the change in overall cell dimensions.

### 5.2. Monoclinic HEWL

Hen egg-white lysozyme (HEWL) is the ‘model’ protein for many studies in protein crystallography (Strynadka & James, 1996 ▸). There are crystal forms in six of the seven crystal systems (Joosten *et al.*, 2014 ▸). The monoclinic form in space group *P*2_1_ has two copies in the asymmetric unit and a monoclinic angle close to 90°. Non-isomorphism can include variations where the monoclinic angle drops below 90°, in which case the convention that the monoclinic angle be greater than 90° means that coincident lattices can be related by a re-indexing operation: the resulting unit cells appear ‘upside-down’ with respect to the monoclinic axis when viewed together. An example of this is found in the structures deposited with PDB codes 2d4k and 1vdp (James Holton, personal communication). *Scotty* identifies this pair of structures as coincident lattices with the appropriate re-indexing operation (Table 2 ▸). The run time, including rigid-body refinement, was 8 s.

### 5.3. Phospholipase A_2_

Snake-venom phospholipase A_2_ (PLA_2_) is synthesized in membrane-bound secretory granules (zymogen granules). The pH and low calcium concentrations in these granules are suboptimal for PLA_2_ activity, allowing it to be safely sequestered in the venom glands before deployment.

There are several seemingly different crystal forms of PLA_2_ deposited in the PDB, which have been discussed in detail elsewhere (Le Trong & Stenkamp, 2007 ▸; McGill *et al.*, 2014 ▸; Bernstein *et al.*, 2020 ▸). PDB entries 1fe5, 1u4j and 1g2x present three alternative unit cells for the same lattice, although a simple unit-cell comparison cannot bring them together. Using PDB entry 1fe5 as the query data, *Scotty* was able to identify the coincident lattices, which represent subgroups of the highest symmetry space group (Table 3 ▸). The run time, including rigid-body refinement, was 8 s.

### 5.4. Concanavalin A

Concanavalin A (ConA), a lectin, undergoes a conformational change that leads to several different crystal forms. The *I*222 form exhibits considerable non-isomorphism (Parkin & Hope, 2003 ▸).

The PDB was queried for experimental structures determined by X-ray crystallography with ‘Concanavalin A’ in their title, in space group *I*222 and with structural similarity to PDB entry 7mg1 (a randomly chosen representative ConA structure in space group *I*222), giving 31 structures. Clustering of unit cells performed using *DBSCAN* on unit-cell parameters (*a*, *b*, *c*) showed two settings: *a* < *c* with 23 entries and *a* > *c* with nine entries. Pairwise NCDist was calculated for all combinations of unit cells after re-indexing as necessary (*a* < *c*). PDB entry 1hqw was identified as the medoid, with unit-cell parameters *a* = 62.6, *b* = 86.6, *c* = 89.3 Å, α = β = γ = 90°, and PDB entry 5wey was identified as the anti-medoid, with unit-cell parameters *a* = 66.6, *b* = 86.6, *c* = 91.8 Å, α = β = γ = 90°. The data for these two structures were extracted from the PDB archive and used as the query in *Scotty*.

**Figure 2:**
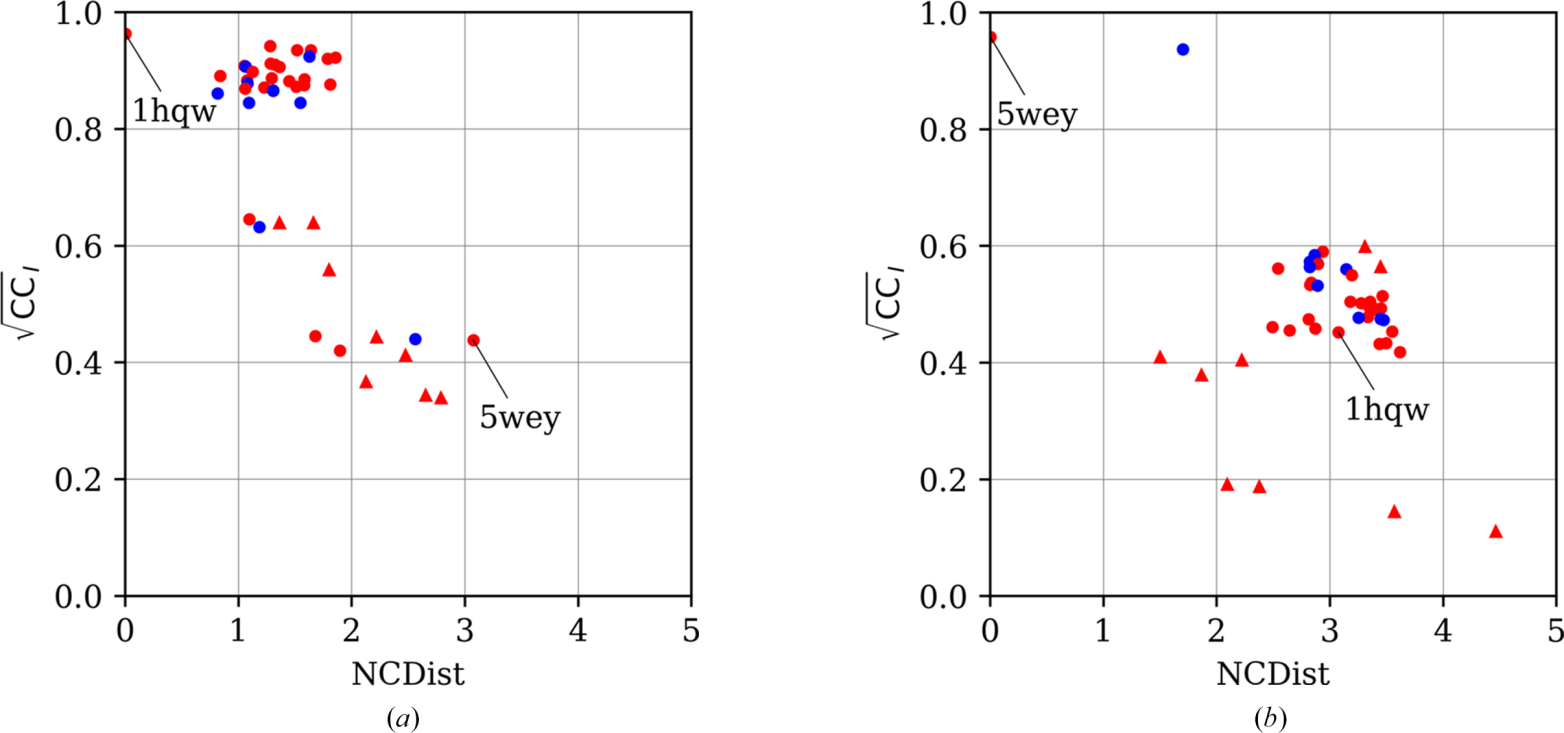
NCDist versus √CC_*I*_ for lattice coincidences in PDB snapshot 20260101 for (*a*) medoid PDB entry 1hqw and (*b*) anti-medoid PDB entry 5wey of 31 selected ConA structures determined in space group *I*222. Colour-coding is by space-group setting: *a* > *b* (red), *a* < *b* (blue). Shape-coding is by sequence identity above/below 95% (circles/triangles). Medoid and anti-medoid points are labelled. The results of the probe with the medoid PDB entry 1hqw returned lower values for NCDist and higher values for √CC_*I*_ than the probe with the anti-medoid PDB entry 5wey.

A scatter plot for NCDist versus √CC_*I*_ for all returned values of NCDist < 5 Å is shown in Fig. 2 ▸. Structures of ConA were returned regardless of setting, together with coincident lattices from homologous proteins. The run time, including rigid-body refinement, was 8 s.

### 5.5. Contaminants

The obsolete PDB entries 4nl6 and 4nl7 were retracted (Seng *et al.*, 2015 ▸) after post-deposition analysis demonstrated that both structures arose from the accidental crystallization of well known *Escherichia coli* contaminants, namely the Gab protein and the Hfq riboregulator (Weiss *et al.*, 2016 ▸). In the former case (PDB entry 4nl6), the identification was made after reanalysis of the deposited diffraction data using *phenix.xtriage* indicated that the originally assigned space group *C*2 was undermerged, with the data merging well in space group *I*422. Searches of the PDB archive based on the remerged *I*422 unit-cell parameters yielded two candidates: d-amino-acid oxidase from *Rhodosporidium toruloides* (PDB entry 1c0i) and the *E. coli* Gab protein (PDB entry 1jr7). Of these, Gab was a far more plausible candidate for accidental crystallization, which was subsequently confirmed by molecular replacement. In the latter case (PDB entry 4nl7), the contaminant was intuited to be a protein with affinity for nickel-chelate columns, leading to the identification of Hfq as a likely candidate; molecular replacement using Hfq confirmed this as the crystallized protein.

Using only the deposited diffraction data from the obsolete PDB entries as the query, *Scotty* was able to identify both contaminants, without requiring remerging of the PDB entry 4nl6 data in *I*422 or any prior assumptions about the identity of the contaminating proteins.

The *C*2 data deposited for PDB entry 4nl6 were matched with PDB entries 1jr7 (released in 2001), 2r6s (released in 2008) and 8cdf (released in 2023), in space group *I*422 (Table 4 ▸).

The *C*2 data deposited for PDB entry 4nl7 were matched with PDB entry 2yht (released in 2011) in space group *P*1 (Table 5 ▸).

## 6. Discussion

Our software *Scotty* can rapidly scan a metric-space index of the PDB archive for closely and distantly related crystal forms and will serve to rapidly phase these structures by DFFT. It should be a standard preliminary analysis, along with other data-analysis methods (*e.g.phenix.xtriage*, *Xtricorder*; McCoy & Read, 2025 ▸).

Lattice coincidences play a role in contaminant screening because many common contaminants crystallize readily and can appear in multiple crystal forms, some of which may be perturbed versions of those deposited in the PDB, making strict space-group and unit-cell matching too brittle for reliable detection (Simpkin *et al.*, 2018 ▸; Long *et al.*, 2008 ▸). By comparing crystal forms using NCDist, contaminant crystals can be recognized even when symmetry assignments differ or cells are distorted or re-indexed. In practice, lattice coincidence analysis broadens the net for identifying contaminants (provided that the crystal form exists in the archive), reducing false negatives.

The *Scotty* procedure for identifying lattice convergences is very sensitive and robust. At the limit of lattice coincidence detection, it is possible that matches are outside the radius of convergence for even wide radius convergence rigid-body refinement. This is made more likely when the lattice coincidences are between macromolecules with low sequence identity. In such cases, it may be necessary to perform molecular replacement to phase the query structure, rather than using DFFT phasing. With knowledge of the lattice-coincident match, the components of the molecular-replacement solution can be put into the coordinates of the matching lattice with *phenix.famos* (Oeffner *et al.*, 2012 ▸).

We aim to extend the use of NCDist-based lattice matching to data reduction during data collection. For this utility, our optimization of the NCDist algorithm for speed will allow an ‘on-the-fly’ tool.

As the PDB archive of structures expands, it may become necessary to revisit the storage of data in the BallTree structure and eliminate low-r.m.s.d. duplicates to increase the efficiency of tree generation and searching. If the archive becomes dominated by ligand screens and high numbers of structures sharing the same deposition group, the query time may tend towards *O*(*N*) and become rate-limiting. Refinements to the calculation of NCDist are likely to continue as computational methods improve. The use of other metrics, and/or optimization of metadata, may also allow us to improve the speed, accuracy and scope of the method.

## Supplementary Material

Niggle cell reduction. DOI: 10.1107/S2059798326005711/nz5021sup1.pdf

## Figures and Tables

**Table 1:** Comparison of lattice coincidences between human prorenin (PDB entry 4amt) with rat prorenin (PDB entry 5mlg) and mouse prorenin (PDB entry 5mkt) The NCDist values are substantially different; however, the √CC_*I*_ metric is only marginally higher for the lower NCDist structure PDB entry 5mkt. PDB entry 4amt can be successfully phased by DFFT methods and wide convergence radius refinement with either structure.

PDB code	Space group	*a* (Å)	*b* (Å)	*c* (Å)	α(°)	β (°)	γ (°)	NCDist to PDB entry 4amt	√CC_*I*_ to PDB entry 4amt
4amt	*I*4_1_	141.3	141.3	75.9	90	90	90	(0.0)	(1.0)
5mlg	*I*4_1_	142.5	142.5	77.0	90	90	90	2.121	0.527
5mkt	*I*4_1_	141.2	141.2	83.0	90	90	90	4.551	0.471

**Table 2:** Comparison of lattice coincidences between HEWL PDB entries 2d4k and 1vdp PDB entry 1vdp is shown under three alternative re-indexing operators, illustrating the effect of re-indexing on identifying lattice coincidences. Although the NCDist value between PDB entries 2d4k and 1vdp is unchanged by re-indexing, the √CC_*I*_ metric varies substantially. The re-indexing that yields the closest lattice coincidence with PDB entry 2d4k is (−*h*, −*k*, *l*).

PDB code	Space group	*a* (Å)	*b* (Å)	*c* (Å)	α (°)	β (°)	γ (°)	Re-index	NCDist to PDB entry 2d4k	√CC_*I*_ to PDB entry 2d4k
2d4k	*P*2_1_	27.2	63.5	59.2	90	92.9	90		(0.0)	(1.0)
1vdp	*P*2_1_	28.0	62.9	60.6	90	90.9	90	*h*, *k*, *l*	1.501	0.398
1vdp	*P*2_1_	28.0	62.9	60.6	90	90.9	90	−*h*, *l*, *k*	1.501	0.202
1vdp	*P*2_1_	28.0	62.9	60.6	90	90.9	90	−*h*, −*k*, *l*	1.501	0.653

**Table 3:** Comparison of lattice coincidences between phospholipase A_2_ PDB entry 1fe5 with PDB entries 1u4j and 1g2x PDB entry 1u4j is shown under two alternative re-indexing operators and PDB entry 1g2x under three re-indexing operations: these re-indexing operators are nonstandard because they involve re-indexing to different unit-cell parameters (standard settings for the different space groups). NCDist values for the other crystal forms are comparable, as are the √CC_*I*_ values for the best re-indexing operator.

PDB code	Space group	*a* (Å)	*b* (Å)	*c* (Å)	α (°)	β (°)	γ (°)	Re-index	NCDist to PDB entry 1fe5	√CC_*I*_ to PDB entry 1fe5
1fe5	*R*32	58.0	58.0	58.0	92.0	92.0	92.0		(0.0)	(1.0)
1u4j	*R*3:*H*	80.4	80.4	99.4	90	90	120	−⅔*h* − ⅓*k* − ⅓*l*, ⅓*h* +⅔*k* − ⅓*l*, ⅓*h* − ⅓*k* − ⅓*l*	2.087	0.753
1u4j	*R*3:*H*	80.4	80.4	99.4	90	90	120	⅓*h* +⅔*k* − ⅓*l*, ⅔*h* +⅓*k* +⅓*l*, ⅓*h* − ⅓*k* − ⅓*l*	2.087	0.137
1g2x	C2	81.0	80.6	57.1	90	90.4	90	*l*, ½*h* + ½*k*, ½*h* − ½*k*	2.080	0.751
1g2x	C2	81.0	80.6	57.1	90	90.4	90	½*h* + ½*k*, *l*, ½*h* + ½*k*	2.080	0.162
1g2x	C2	81.0	80.6	57.1	90	90.4	90	½*h* + ½*k*, *l*, ½*h* − ½*k*	2.080	0.158

**Table 4:** Comparison of lattice coincidences for obsolete entry PDB entry 4nl6, the contaminant protein *E. coli* Gab, with non-obsolete PDB entries 1jr7, 2r6s and 8cdf

PDB code	Space group	*a* (Å)	*b* (Å)	*c* (Å)	α (°)	β (°)	γ (°)	Re-index	NCDist to PDB entry 4nl6	√CC_*I*_ to PDB entry 4nl6
4nl6	*C*2	137.0	169.8	108.8	90	128.5	90		(0.0)	(1.0)
8cdf	*I*422	121.1	121.1	136.5	90	90	90	*l*, *h* − *k*, ½*h* + ½*k* − ½*l*	1.969	0.892
2r6s	*I*422	120.9	120.9	137.2	90	90	90	*l*, *h* − *k*, ½*h* + ½*k* − ½*l*	1.840	0.884
1jr7	*I*422	120.5	120.5	136.6	90	90	90	*l*, *h* − *k*, ½*h* + ½*k* − ½*l*	1.375	0.882

**Table 5:** Comparison of lattice coincidences for obsolete PDB entry 4nl7, the contaminant protein *E. coli* Hfq riboregulator, with non-obsolete entry 2yht

PDB code	Space group	*a* (Å)	*b* (Å)	*c* (Å)	α (°)	β (°)	γ (°)	Re-index	NCDist to PDB entry 4nl7	√CC_*I*_ to PDB entry 4nl7
4nl7	*C*2	107.1	62.3	57.1	90	95.7	90		(0.0)	(1.0)
2yht	*P*1	61.2	61.2	53.1	82.6	87.3	60.0	*h* − 2*k*, *h*, *l*	2.338	0.583

## Data Availability

*Scotty* will be made available through the *Phenix* and *CCP*4 software packages.

## Appendix A Speed optimization of NCDist

NCDist calculations are the main computational bottleneck for BallTree traversal and a significant computational bottleneck for database generation. The complexity of the algorithm lies in the requirement to account for Niggli-cell boundary crossing, which is a combinatorial problem. The algorithm is not exhaustive in its examination of all combinations, but instead accounts for crystallographically relevant cases, thereby avoiding the need to examine lattices that are crystallographically implausible for macromolecules of realistic dimensions.

The code for NCDist used for metric-space indexing is derived from the *SAUC* server code distributed on GitHub (https://github.com/yayahjb/sauc). We optimized this code for computational speed, achieving a speed improvement of two orders of magnitude, thereby bringing database generation down to minutes and search down to less than a second.

The original code used C-style macros rather than functions, having been written when C compilers could not reliably inline. Modern compilers perform aggressive inlining of functions and sophisticated register allocation, using the type information and semantic structure that macros discard. The use of macros now inhibits these native, build-specific compiler optimizations, which alone provide significant performance gains. Refactoring macro-based code is beneficial not only for speed but also long-term code maintenance, since macros give poor compile-time and run-time diagnostics.

Further speed-up in NCDist came from standard algorithmic refactoring of the code. Operations that were previously branch-heavy and recomputed many times were replaced by a table-driven formulation, where boundary projectors and sign-change transforms are now stored as static constant matrices and accessed via simple index lookups rather than being rebuilt on the fly. The distance calculation in **G**^6^ was tightened by consolidating quadratic-form evaluations, eliminating redundant inner products and inlining the critical paths so the compiler can optimize them aggressively. Data structures were laid out as contiguous arrays to improve cache locality, and simple early-exit logic now avoids unnecessary boundary exploration once a minimal through-boundary distance has already been established. Optionally, independent floating-point operations may be vectorized by compiling with xsimd, which itself gives a factor of two speedup. The resulting NCDist calculation was numerically almost identical to the starting algorithm (within tiny floating-point tolerances). In rare cases, tiny floating-point differences during the calculation can lead to different decisions at boundaries. Usually, the combinatorial path subsequently restores the NCDist to within rounding errors; however, these differences can also randomly improve or degrade the NCDist.

## Appendix B Metric-space index generation

### b1. BallTree

We organized the Niggli-reduced unit cells for the PDB into a ‘BallTree’ structure using the Python library scikitlearn (Pedregosa *et al.*, 2011 ▸). A ‘BallTree’ is a hierarchical metric-space index that recursively partitions a dataset into nested hyper-spherical regions (‘balls’), each defined by a centroid and radius that enclose the points within that node. Because partitioning relies only on pairwise distances, the data structure can support any metric that satisfies the triangle inequality and remains effective even in non-Euclidean spaces: although NCDist is derived from a Euclidean metric, the geometry is highly irregular because neighbourhoods are fragmented by the Niggli boundaries.

We used a leaf size of 2 in ‘BallTree’ generation, empirically optimized for minimizing search time. A leaf size of 2 minimizes the number of distance computations performed at the leaf level, producing tight terminal nodes that enable aggressive pruning through the triangle inequality. While this results in a deeper tree, the additional traversal overhead is negligible compared with the reduction in NCDist evaluations, yielding faster searches than larger leaf sizes, while remaining substantially more efficient than brute-force pairwise comparisons. This behaviour mirrors the design of NearTree in *SAUC*, which similarly favours small terminal node sizes.

In our application, *k* ≪ *N*, where *k* is the number of returned neighbours (normally between 1 and 100) and *N* is the size of the index (200 000 PDB entries), and the metric evaluation (NCDist) is costly. ‘BallTree’ is *O*(*N*log*N*) nearest-neighbour queries for build and approximately *O*(log*N* + *k*) to traverse, with *k* ≪ *N*.

### b2. Duplicate metadata

The Protein Data Bank contains structures that can be considered duplicates, in that the space groups are identical and the macromolecular components, unit cells and deposited atomic coordinates are practically identical. The family of tetragonal HEWL is the archetype for such structures.

For this purpose, the internal variation within each duplicate group must be tightly bounded: the differences between members of a duplicate set must be much smaller than the tolerances when matching a query against the index. Any members of a duplicate group must be effectively interchangeable within the parameters of the search, so that selecting a single representative does not mask differences that would have been informative. Otherwise, the collapsing of duplicates will induce false negatives in the search.

Duplicates are defined very narrowly in a three-pass procedure.

Firstly, possible duplicates were identified by matching crystallographic parameters: identical space groups and space-group settings, unit cells within tolerances of 1.5 Å on each of *a*, *b*, *c* and 1.5° in each of α, β, γ and an overall NCDist < 1.0. This selects not just for identical lattices but more strictly for the same deposited description of the crystallographic lattice. Unit-cell differences of 1.5 Å mean that the data are correlated at 3 Å, the resolution for √CC_*I*_ calculation, even in the direction of greatest difference in sampling (Fig. 1 ▸). Rotations are more difficult to quantify in terms of correlations; however, a 1.5° rotation results in a 1.5 Å shift of atoms at a distance of 60 Å from the rotation axis.

Secondly, this list was pruned to duplicates containing identical (or nearly identical) macromolecules by requiring identical ‘primary’ sequences – no point mutations, insertions or deletions, although end gaps were permitted, including leading or trailing tags – so that the effective sequence identity was 100%, preventing any false negatives due to sequence mismatches. Note that the sequences associated with an entry are normally the sequences of the proteins in the UniProt database and therefore they do not normally record any of the modifications due to site-directed mutagenesis that may be present in the structure itself.

On a third pass, duplicates were retained if their r.m.s.d. without any fitting was less than 2 Å for all atoms, which is more restrictive than the r.m.s.d. of 2.0 Å over main-chain coordinates established as the quantitative boundary for defining highly similar protein structural folds (Carugo & Pongor, 2001 ▸). The r.m.s.d. was calculated with *phenix.superpose_pdbs*, which calculates the r.m.s.d. for either protein, nucleic acid or mixed protein/nucleic acid structures. The calculation of r.m.s.d. was deemed to be unnecessary if the reference and duplicate were in the same deposition group: these duplicates were retained without a third pass.

The efficiency of BallTree-based tree construction and nearest-neighbour searches is reduced in the presence of many duplicate points because the underlying partitioning strategy assumes good spatial separation between data points. Duplicates lead to poorly discriminating splits and a loss of pruning efficiency during queries. However, since neither tree construction nor querying were a practical bottleneck in the workflow, and the return of all PDB matches is of interest for later analysis, the number of data points stored in the BallTree itself was not reduced after this analysis.

### b3. Generation time

The initial duplicate scan was overwhelmingly rate-limiting for metadata generation: the duplicate scan required over 3.6 million pairwise r.m.s.d. calculations, each requiring loading of the data from disk. These calculations were executed using shared-memory parallelism across available CPU cores. Updates to the PDB NCDist metric-space index to synchronize to the PDB archive require only incremental updates to the pairwise r.m.s.d. calculations, at much less computational cost.

The process of PDB NCDist metric-space index regeneration from the PDB archive, using stored pairwise r.m.s.d. calculations, took minutes: this includes the time for extraction of crystallographic entries from the PDB archive, extraction of summary *PDB_REDO* data from the *PDB_REDO* server, processing of metadata, generating the ‘BallTree’ metric-space index and unit tests.

### b4. Duplicate search space

Entries in the PDB metric-space index were clustered into isomorphous groups by building an undirected graph where the edges represent the duplicates and then finding all connected components. There were 161 121 clusters of replicate entries.
