# Supplementary material for: Scotty: lattice coincidences for macromolecular crystallographic phasing
Source: Acta Crystallogr D Struct Biol. 2026 Jun 24;82(Pt 7):813–23. doi: 10.1107/S2059798326005711 (PMC13317677; doi:10.1107/S2059798326005711)
Supplement: Supplementary file 1 [file d-82-00813-sup1.pdf]

STRUCTURAL BIOLOGY

Volume 82 (2026)

Supporting information for article:

Scotty: lattice coincidences for macromolecular crystallographic phasing

Airlie J. McCoy, Lawrence C. Andrews, Herbert J. Bernstein and Randy J. Read

S1. Niggli Cell Reduction

To qualify as a Niggli reduced cell, the lattice basis must satisfy two primary sets of inequalities regarding shortest vector and angle conditions. If the metric tensor lies on the boundary of the Niggli cone (e.g., when lengths are equal or angles reach geometric limits), additional tie-breaking rules are applied to make the resulting cell description completely unique. The additional boundary rules define how ties are resolved when the metric tensor lies on a boundary of the Niggli cone. Thus, when A = B or B = C, the relevant off-diagonal terms are ordered by absolute value; when $|\xi|$, $|\eta|$, or $|\zeta|$ reaches its limiting value relative to the basis lengths, further inequalities involving the remaining off-diagonal terms are imposed. These rules remove otherwise equivalent reduced descriptions and guarantee that the resulting Niggli cell is strictly unique.

S1.1. Basis Vectors and the Metric Tensor

For a given set of primitive basis vectors a, b, and c, the cell parameters are defined by their squared lengths and scaled dot products:

$A = a \cdot a = a^2$

$B = b \cdot b = b^2$

$C = c \cdot c = c^2$

$\xi = 2(b \cdot c) = 2bc \cos \alpha$

$\eta = 2(a \cdot c) = 2ac \cos \beta$

$\zeta = 2(a \cdot b) = 2ab \cos \gamma$

The corresponding metric tensor G, which fully describes the lattice metrical properties, is defined as:

$$G = \begin{bmatrix} A & \frac{1}{2}\zeta & \frac{1}{2}\eta \\ \frac{1}{2}\zeta & B & \frac{1}{2}\xi \\ \frac{1}{2}\eta & \frac{1}{2}\xi & C \end{bmatrix}$$

S1.2. Main Inequalities (Shortest Vector Conditions)

These ensure that the chosen basis vectors are the three shortest non-coplanar translation vectors of the lattice:

$A \leq B \leq C$

$|\xi| \leq B$, $|\eta| \leq A$, $|\zeta| \leq A$

S1.3. Sign Inequalities (Angle Conditions)

These classify the cell into one of two distinct types based on the signs of the interaxial angles. All dot products must be strictly positive (Type I, all acute angles) or strictly non-positive (Type II, all obtuse or right angles):

Either:

$\xi > 0, \eta > 0, \zeta > 0$ (Type I)

Or:

$\xi \leq 0, \eta \leq 0, \zeta \leq 0$ (Type II)

S1.4. Equality of Squared Lengths:

If two basis vectors have identical lengths, their corresponding off-diagonal terms are ordered by absolute value:

$A = B \rightarrow |\xi| \leq |\eta|$

$B = C \rightarrow |\eta| \leq |\zeta|$

S1.5. Limiting Values of Off-Diagonal Terms:

When an off-diagonal term reaches its upper or lower limiting bound, further inequalities involving the remaining terms are imposed:

$\xi = B \rightarrow |\xi| \leq |\eta|$

$\eta = A \rightarrow |\zeta| \leq |\xi|$

$\zeta = A \rightarrow |\eta| \leq |\xi|$

$\xi = -B \rightarrow \zeta \geq 0$

$\eta = -A \rightarrow \zeta \geq 0$

$\zeta = -A \rightarrow \eta \geq 0$

S1.6. The Krivý–Gruber Algorithm Conditions

The Krivý–Gruber algorithm enforces an additional diagonal condition to ensure the body diagonal a+b+c is not shorter than c.

If

$A + B + C + \xi + \eta + \zeta < 0$

then a reduction transformation must be applied (typically updating the basis vectors to reduce the diagonal length).

On the equality boundary, where the metric tensor satisfies:

$A + B + C + \xi + \eta + \zeta = 0$

The algorithm breaks the tie and ensures uniqueness by requiring:

$2A + 2\eta + \zeta \geq 0$
